# Changes in Myocardial Composition and Conduction Properties in Rat Heart Failure Model Induced by Chronic Volume Overload

**DOI:** 10.3389/fphys.2016.00367

**Published:** 2016-08-25

**Authors:** David Sedmera, Jan Neckar, Jiri Benes, Jana Pospisilova, Jiri Petrak, Kamil Sedlacek, Vojtech Melenovsky

**Affiliations:** ^1^Institute of Physiology, Czech Academy of SciencesPrague, Czech Republic; ^2^First Faculty of Medicine, Institute of Anatomy, Charles University in PraguePrague, Czech Republic; ^3^Institute of Clinical and Experimental MedicinePrague, Czech Republic; ^4^Department of Radiology, First Faculty of Medicine, Charles University in PraguePrague, Czech Republic; ^5^First Faculty of Medicine, Institute of Pathological Physiology, Charles University in PraguePrague, Czech Republic; ^6^First Faculty of Medicine, Biotechnology and Biomedicine Center of the Academy of Sciences and Charles University in VestecVestec, Czech Republic

**Keywords:** connexin43, autonomic heart innervation, hypertrophy, conduction velocity, aorto-caval fistula

## Abstract

Volume overload leads to development of eccentric cardiac hypertrophy and heart failure. In our previous report, we have shown myocyte hypertrophy with no fibrosis and decrease in gap junctional coupling via connexin43 in a rat model of aorto-caval fistula at 21 weeks. Here we set to analyze the electrophysiological and protein expression changes in the left ventricle and correlate them with phenotypic severity based upon ventricles to body weight ratio. ECG analysis showed increased amplitude and duration of the P wave, prolongation of PR and QRS interval, ST segment elevation and decreased T wave amplitude in the fistula group. Optical mapping showed a prolongation of action potential duration in the hypertrophied hearts. Minimal conduction velocity (CV) showed a bell-shaped curve, with a significant increase in the mild cases and there was a negative correlation of both minimal and maximal CV with heart to body weight ratio. Since the CV is influenced by gap junctional coupling as well as the autonomic nervous system, we measured the amounts of tyrosine hydroxylase (TH) and choline acetyl transferase (ChAT) as a proxy for sympathetic and parasympathetic innervation, respectively. At the protein level, we confirmed a significant decrease in total and phosphorylated connexin43 that was proportional to the level of hypertrophy, and similarly decreased levels of TH and ChAT. Even at a single time-point, severity of morphological phenotype correlates with progression of molecular and electrophysiological changes, with the most hypertrophied hearts showing the most severe changes that might be related to arrhythmogenesis.

## Introduction

Heart failure is a pathological state in which the heart is unable to pump blood sufficiently to supply the organism with oxygen and nutrients. It could be due to either abnormal functional demands (pressure or volume overload), or intrinsic problems within the myocardium (inflammation, tumor, ischemic cardiomyopathy). Independent of its etiology, it decreases patient's quality of life and increases the risk of sudden death (reviewed in Stevens et al., 2013). Several large outcome studies in heart failure patients showed a fivefold increase in the risk of sudden death (Kannel et al., 1988) and low left ventricular ejection fraction and incidence of ventricular tachycardia on Holter monitoring as predictors of both overall mortality and sudden cardiac death (Gradman et al., 1989). One of the mechanisms was suggested to be changes in cardiac repolarization (Tomaselli et al., 1994). A significant decrease in quality of life of heart failure patients was demonstrated in community settings (Hobbs et al., 2002), and health-related quality of life was found to independently predict mortality and hospitalization after adjustment for ejection fraction, age, and functional classification (Konstam et al., 1996).

In case of volume-overload heart failure, the increased volume of circulated blood worsens myocardial function and is compensated by eccentric hypertrophy with possible dilation followed by concentric hypertrophy (Ford, 1976). When these initial compensation mechanisms are exhausted, the decompensated heart failure sets in (Hood et al., 1968).

While volume-overload heart failure is not the most common cause of cardiac decompensation in the patient population, there are situations such as chronic dialysis shunt, arterio-venous malformation, or valve regurgitation that could lead to to ventricular failure. Arteriovenous fistula was found to be the third most common cause (23%, *N* = 120) of high-output heart failure in a recent (2010–2014) clinical study performed at the Mayo clinic (Reddy et al., 2016). However, mitral regurgitation (which does lead to volume overload) was implicated as an aggravating factor in up to 30% of heart failure cases of other etiologies. The study by Grigioni et al. (1999) have demonstrated that mitral valve prolapse, while leading to mitral regurgitation, is also associated with sudden death. The proportion of sudden cardiac deaths attributable to arrhythmias is uncertain; a recent review aimed at optimization of guidelines for implantation of cardioverter-defibrillator suggests that both cardiac hypertrophy and low ejection fraction are contribution factors (Stevens et al., 2013), and further research exploring the links between genetic and environmental factors leading to transition from cardiac hypertrophy to heart failure is warranted.

Arrhythmias are a common complication of heart failure. They are often linked to increased electrical heterogeneity of the myocardium and slowed impulse conduction (Shah et al., 2005). The mechanisms responsible for arrhythmogenicity are myocardial fibrosis, changes in membrane excitability or tissue architecture and alterations in expression of connexins (Libby et al., 2008).

Several animal models of heart failure with different etiology were created (Akar and Tomaselli, 2005). Ischemic cardiomyopathy can be induced by creation of myocardial infarction by coronary artery ligation. Pressure overload is induced by transverse aortic constriction, renal artery occlusion (via activation of renin-angiotensin system) or in spontaneous hypertension (SHR rat model). Eccentric hypertrophy could be induced experimentally by either cardiac tachypacing (Akar et al., 2004) or volume overload caused by arteriovenous shunt (Hatt et al., 1979; Melenovsky et al., 2011); the later models simulate human aortic insufficiency, or arterio-venous shunt in chronically dialyzed patients.

Using the rat volume overload model created by aorto-caval fistula (ACF), numerous insights into pathogenesis of volume-overload induced heart failure were obtained. Volume overload leads to increased heart weight due to biventricular eccentric hypertrophy (Benes et al., 2011a). Unlike in pressure-overload hypertrophy, there is a remarkable lack of increased fibrosis (Ryan et al., 2007). There is also a decrease in the amount of connexin43 protein and its phosphorylation (Benes et al., 2011a). Metabolic changes in this model indicate changed energetics with increased lipolysis and attenuated response to insulin (Melenovsky et al., 2011). At the level of cardiac mechanics, there is a decrease in left ventricular fractional shortening, decreased slope in pressure-volume relationship indicating systolic dysfunction that is apparent also at the level of isolated myocytes (Guggilam et al., 2012). There is also abnormal calcium handling and attenuated *in vivo* response to beta-adrenergic stimulation (Guggilam et al., 2012).

Further indirect evidence that lethal arrhythmias occur in this model stems from the long-term survival study (Melenovsky et al., 2012), which showed two types of death, one due to heart failure with edemas, and the other being sudden cardiac death, which is widely regarded as due to arrhythmias. These results are corroborated by reports from other volume overload animal models such as dog (Peschar et al., 2003) or rabbit (van Borren et al., 2012). Arrhythmias are known to be associated with cardiac hypertrophy also in the clinical scenario (reviewed in Stevens et al., 2013), and heart failure defined as low (< 35%) ejection fraction of the left ventricle represents an additive risk factor for sudden cardiac death. However, the exact mechanisms of this relationship are not completely understood, which prompted us to further explore possible pathogenetic changes in the rat ACF model.

All the factors combining to create an arrhythmogenic substrate, which increases the incidence of potentially lethal arrhythmias, were also reported in this model (Benes et al., 2011a). Longitudinal analysis showed that most deaths occur between 28 and 50 weeks after shunt creation, and the most hypertrophied heart are prone to sudden death (Melenovsky et al., 2012). Increased heart weight was inversely associated with the length of survival. We have therefore set to investigate if and how the level of cardiac hypertrophy correlates with conduction anomalies and molecular changes and to explore which of these parameters could be used for monitoring of progression from compensated hypertrophy to end-stage heart failure. We hypothesized that subdividing the experimental group by phenotype severity would uncover parameters that change during transition from compensated hypertrophy to heart failure.

## Methods

### Animals

The rats were kept in air-conditioned animal facility of the Institute of Clinical and Experimental Medicine on a 12-/12-h light/dark cycle. Throughout the experiments, the rats were fed a standard diet (0.45% NaCl, 19–21% protein) supplied by SEMED (Prague, Czech Republic) and had free access to tap water. The experiments were performed according to applicable law (311/1997), were approved by the Ethic Committee of the Institute of Clinical and Experimental Medicine (Prague, Czech Republic, approval number 305/09/1390), and conform to the guidelines from Directive 2010/63/EU of the European Parliament on the protection of animals used for scientific purposes. The ACF was created under general anesthesia of ketamine and midazolam as described (Benes et al., 2011a; Melenovsky et al., 2012). Briefly, the abdominal aorta was pierced with a 1.2 mm needle into the inferior vena cava. The needle was removed after clamping the aorta above and applying acrylamide tissue glue. The shunt functionality was verified after 3 min by pulsation of the inferior vena cava. Sham-operated animals underwent the same procedure without puncture. Male Wistar rats weighting 300–350 g were used for these experiments and the changes were evaluated after 21 weeks unless stated otherwise. For quantitative analysis we used a total of 20 ACF rats and 10 sham-operated controls. The parameters and allocation to different severity groups (mild, moderate, and severe) according to heart to body weight ratio (HBWR, heart weight being defined as sum of both ventricles and the septum) are listed in Table 1. Division into three sub-groups (of size permitting reasonable statistical testing) was performed to evaluate any correlation between the severity of hypertrophy and other markers of cardiac hypertrophy or failure.

**Table 1:** **Summary of averages of all measured parameters by group (according to HBWR)**.

**Parameter**	**Sham (*n* = 10)**	**ACF mild (*n* = 5)**	**ACF moderate (*n* = 8)**	**ACF severe (*n* = 4)**	**ANOVA**
**ORGAN WEIGHTS**					
Body weight (BW), *g*	578 ± 48	610 ± 46	581 ± 48	595 ± 45	0.7
Heart weight (HW), *g*#	1.65 ± 0.18	2.90 ± 0.20^**^	3.16 ± 0.20^**^	3.85 ± 0.19^**^	< 0.0001
HW/BW ratio, *g/kg*	2.87 ± 0.33	4.75 ± 0.14 ^**^	5.45 ± 0.21 ^**^	6.4 ± 0.38 ^**^	< 0.0001
LV, *g*	1.03 ± 0.15	1.74 ± 0.08 ^**^	1.95 ± 0.19 ^**^	2.27 ± 0.24 ^**^	< 0.0001
Septum, *g*	0.31 ± 0.09	0.54 ± 0.08 ^*^	0.56 ± 0.79 ^*^	0.70 ± 0.05 ^*^	< 0.0001
RV, *g*	0.31 ± 0.03	0.62 ± 0.04 ^**^	0.65 ± 0.11 ^**^	0.93 ± 0.10 ^**^	< 0.0001
RV/LV ratio^§^	0.23 ± 0.03	0.27 ± 0.01	0.26 ± 0.03 ^*^	0.32 ± 0.02 ^**^	0.0002
Lungs, *g*	1.91 ± 0.06	2.42 ± 0.40	2.59 ± 0.42 ^*^	3.50 ± 0.40 ^**^	< 0.0001
Lungs/BW, *g/kg*	3.40 ± 0.60	3.95 ± 0.58	4.46 ± 0.61 ^**^	5.84 ± 0.55 ^**^	< 0.0001
**ELECTROCARDIOGRAM**					
Heart rate, *bpm*	414 ± 39	415 ± 38	397 ± 40	403 ± 36	0.8
P amplitude, μ*V*	40 ± 27	80 ± 20	80 ± 29 ^*^	61 ± 28	0.05
P duration, *ms*	15 ± 1.5	19 ± 1.4 ^**^	18 ± 1.6 ^**^	18 ± 1.4 ^**^	0.0003
PR duration, *ms*	47 ± 3	55 ± 4 ^**^	52 ± 3^*^	58 ± 3 ^**^	0.0008
QRS duration, *ms*	18 ± 1.2	21± 1.4 ^**^	21 ± 1.3 ^**^	22 ± 1.2 ^**^	0.0002
QT duration, *ms*	78 ± 9	82 ± 8	80 ± 8	77 ± 10	0.9
QRS amplitude sum, μ*V*	386 ± 210	569 ± 200	591 ± 209	718 ± 190	0.08
T amplitude, μ*V*	70 ± 36	54 ± 38	47 ± 37	− 10 ± 47 ^*^	0.05
ST height, μ*V*	16 ± 63	−34 ± 60	−33 ± 61	− 121 ± 57 ^**^	0.02
**OPTICAL MAPPING**					
APD50, *ms*	56 ± 12	69 ± 12	75 ± 13^*^	65 ± 12	0.04
APD90, *ms*	94 ± 21	101 ± 20	121 ± 21	97 ± 19	0.07
CV min, *cm/s*	14.6 ± 11.1	32 ± 7.4^*^	13.7 ± 7.7	12.3 ± 7.1	0.004
CV max, *cm/s*	102 ± 72	184 ± 48	136 ± 50	102 ± 47	0.09
Anisotropy	7.1 ± 6.6	6.3 ± 6.0	12.3 ± 12.4	8.9 ± 5.9	0.4

Values are means ± SD, ANOVA, Dunnet test

* p < 0.05 and

** p < 0.01 vs. sham controls.

# HW, sum of botd ventricles and septum;

§ LV, LV weight + septum weight; CV, conduction velocity, APD, action potential duration; ACF, aorto-caval fistula; BW, body weight; LV, left ventricle; RV, right ventricle.

### ECG analysis

**Figure 1:**
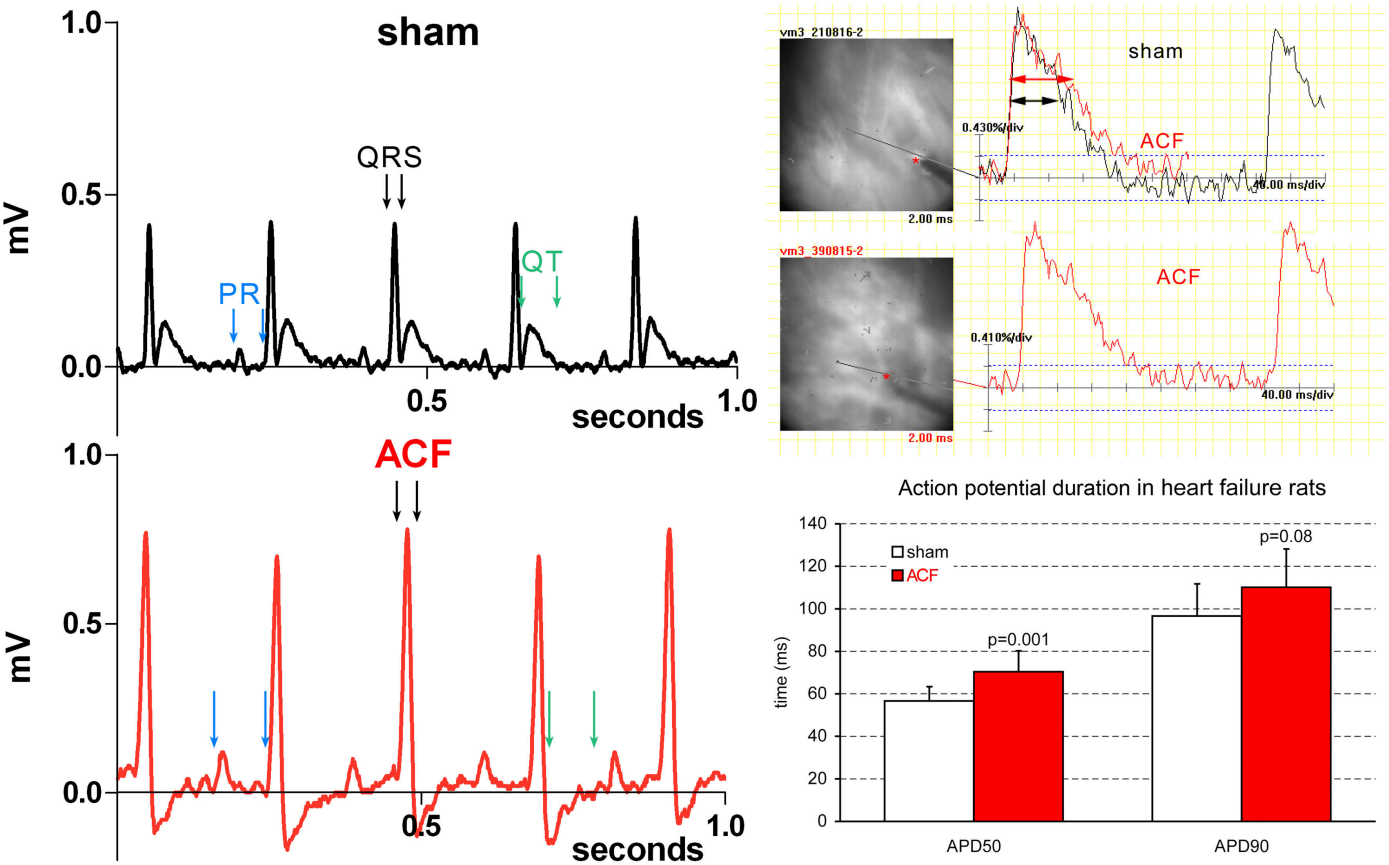
**Representative ECG and EP recordings highlighting the changes in quantitative parameters**. Note an increased P wave amplitude and duration, increased R amplitude, and prolonged QRS duration in a typical ACF recording. Prolongation of APD50 (right column) is present in the ACF group as whole (compare with the sub-group values in Table 1).

Rats were anesthetized with an intraperitoneal injection of ketamine/midazolam (50/5 mg/kg). Four ECG leads were attached at the proximal part of the limbs using clips attached to subcutaneous needles. ECG recording was performed using FE 132 Bioamp and Powerlab 8 (ADInstruments, Australia) data acquisition tool with 1 kHz sampling. Recordings were off-line analyzed using LabChart Pro 7 program with ECG module, with built-in algorithm for wave detection specific to rodents. Successive 200 beats were extracted from the LabChart channel from the lead with the best signal to noise ratio (lead aVR). The beats labeled as good by the classifier were averaged using alignment to the QRS maximum to form a single beat for analysis (Figure 1). The R wave was identified by the program as the most positive value in the neighborhood of the Beat Marker. The start and end of the QRS complex were determined by searches on each side of the R wave for regions where the slope (dV/dt) falls to sufficiently low values. Isoelectric level was defined as the median of all data values preceding the QRS complex. Absolute values of Q, R, and S were added to calculated QRS sum voltage. The peak of the P wave was defined as the point of the greatest absolute deviation from the isoelectric line in the interval from pre-P to just before the beginning of QRS. The beginning of P wave was determined by intersection of the isoelectric line and a straight line fitted by least squares to points 15–60% prior of the peak of P wave. T wave was defined as the first significant peak of either sign, starting from the point after the end of QRS. The end of T was defined by the first return to isoelectric level. ST height was arbitrarily measured 15 ms after QRS alignment point. QT interval was corrected for heart rate (QTc) using the Bazett formula.

### Optical mapping

The animals were anesthetized with sodium pentobarbital (60 mg/kg) and 100 units of heparin per animal were administered to prevent blood clotting. They were then weighted and euthanized by cervical dislocation and their hearts were rapidly excised and cannulated via the ascending aorta on ice in Tyrode's solution bubbled with 100% O_2_ (composition: NaCl 145 mmol/l, KCl 5.9 mmol/l, CaCl_2_ 1.1 mmol/l, MgCl_2_ 1.2 mmol/l, glucose 11 mmol/l, HEPES 5 mmol/l; pH = 7.4). Lungs were dissected and weighted as well. After stabilization in a horizontal Langendorff perfusion bath at 37°C (Radnoti, Inc) at a perfusion rate of 10 ml/g/min, the hearts were bolus stained with a mixture of 200 μl of 0.125% di-4-ANNEPS in DMSO (Invitrogen) and 50 μl of 0.417% blebbistatin (Sigma, motion inhibitor drug) injected into a compliance chamber of the system (de la Rosa et al., 2013). Imaging was performed using the ULTIMA L camera (SciMedia, Japan) under a 2x, 0.14NA objective lens (effective pixel size 80 micrometers) with water immersion lens cap (Olympus, Japan) on a fixed-stage BX51 WI epifluorescence microscope (Olympus, Japan) equipped with a 150 W Xe arc lamp (Cairn, UK) and an appropriate wide green filter set. Images were acquired at 333, 500, and 1000 frames per second; for consistency and optimal signal to noise ratio, data sets acquired at 500 frames per second were chosen for analysis. The hearts were electrically paced from the middle of the left ventricular free wall at 300 ms cycle length (5 mA, 2 ms pulse width). This was possible since the blebbistatin at the concentration necessary to inhibit contractions considerably slowed down the intrinsic heart rate. Imaged area included a square of 8 × 8 mm in the middle of left ventricular free wall (lateral view) where the orientation of the subepicardial layer of cardiomyocytes is predominantly oblique (Morley and Vaidya, 2001).

### Analysis of recordings

**Figure 2:**
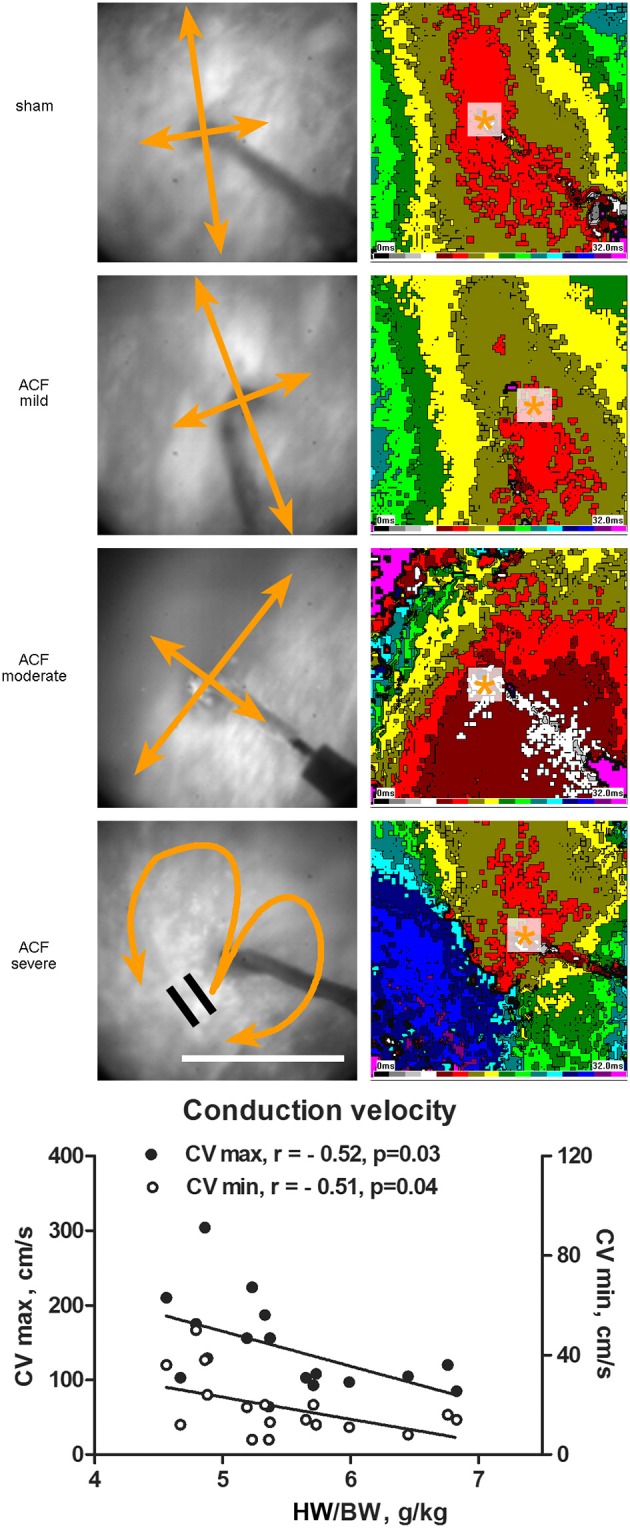
**Epicardial activation patterns of the left ventricle during electrical pacing**. Representative activation maps constructed at 2 ms intervals from the LV mid-portion lateral wall (field of view 8 × 8 mm) are shown for each group. Pacing cycle length is 300 ms in all cases. Asterisk indicates the site or pacing, bidirectional arrows the direction of maximal and minimal conduction velocity. Scale bar 5 mm. The graph below shows correlation between the maximal and minimal conduction velocity and phenotype severity. r, Pearson's correlation coefficient. Line represents linear regression.

The data was band-pass filtered and processed using a 3 × 3 median filter to reduce noise. Action potential duration at 50 and 90% of amplitude was then measured from the recordings in several representative subregions of each heart. The first derivative was then numerically calculated, and its peak was used to detect pixel activation time. Spatio-temporal activation maps (Figure 2) were then constructed in the BV_Ana software (SciMedia, Japan). Using this software, longitudinal (maximal) and transverse (minimal) conduction velocity was measured, along the prevailing long and short axis of subepicardial myocytes as defined in rodents (Morley and Vaidya, 2001). Anisotropy was defined as the ratio between the transverse (CVmin) and longitudinal (CVmax) conduction velocity.

### Western blotting

Left ventricular free wall samples were carefully weighted and then immediately frozen in Eppendorf tubes in liquid nitrogen. They were then pulverized (*N* = 3 per group) under liquid nitrogen and lysed in NHT buffer (140 mM NaCl, 10 mM HEPES, 1.5% Triton X-100, phosphatase inhibitor cocktail, pH 7.4). Lysates were cleared by centrifugation at 14000 × g. Protein concentration was determined by the Bradford assay (Bio-Rad, CA, USA). Each sample (40 μg) was combined with SDS loading buffer containing DTT, boiled for 5 min and resolved by 10%SDS-PAGE in Tris-Glycine buffer. Electrophoresis was performed at constant voltage for 30 min at 45 V per gel, and then at 90 V per gel until the dye front reached the gel bottom. Proteins were then transferred to 0.45 μm PVDF membranes (Millipore, MA, USA) in a semi-dry blotter (Trans-Blot Turbo, Bio-Rad, CA, USA) at constant current and voltage (1.0 A, 25 V) for 30 min. Membranes were incubated with blocking buffer containing PBS, 0.1% TWEEN 20 and 5% non-fat dried milk for 1 h. As primary antibodies, rabbit anti-connexin43 (1:6000, Sigma-Aldrich, MO, USA), rabbit anti-phospho-connexin43 (1:1000, Cell Signaling Technology, MA, USA), goat anti-choline acetyltransferase (1:1000, Millipore, MA, USA) and rabbit anti-tyrosine hydroxylase (1:1000, Sigma-Aldrich, MO, USA) were used. Rabbit anti-GAPDH antibody (1:10000, Sigma-Aldrich, MO, USA) was used as a loading control. After thorough washing in blocking buffer a secondary horseradish peroxidase-conjugated anti-mouse or anti-rabbit antibody was applied for 1 h (1:10,000, Sigma-Aldrich, MO, USA). After washing, signal was detected using Western Blotting Luminol Reagent (Santa Cruz Biotechnology, CA, USA) and membranes were exposed to X-ray films (Kodak, NY, USA). Developed films were scanned on GS-800 calibrated densitometer (Bio-Rad, CA, USA) and the signal was quantified using Quantity One software (Bio-Rad, CA, USA).

### Statistical analysis

All the results are expressed as mean ± SD. Data were assembled and statistically analyzed in JMP10 software (SAS, USA) using *t*-test (for between group differences) or ANOVA and Dunnet *post-hoc* test for comparison of means of subgroups compared to control group. In addition, we divided the ACF animals into two groups (milder and more severe) based on lung to body weight ratio to distinguish between compensated hypertrophy and overt left ventricular failure. Pearson's correlation coefficient was used for assessment of correlation between continuous variables. *P*-value less than 0.05 was considered significant.

### Images

Plates were finally assembled and labeled in Adobe Photoshop (v. 8.0, Adobe Systems, Palo Alto, CA, USA).

## Results

Table 1 lists all the variables measured in experimental animals with their allocation into different experimental subgroups. Two ACF animals died prior to study termination; autopsy performed in one revealed increased heart weight and grossly increased lung weight with presence of ascites and hydrothorax, suggesting heart failure as the cause of death. One ACF animal with no change in the HBWR and normal lung weight at the time of sampling was excluded from the analysis. The fistula was patent, but hemodynamically insignificant, a situation that occus in less than 5% of the operated animals. According to HBWR, the ACF animals were arbitrarily subdivided for quantitative sub-group analysis into mild (range 4.55–4.88, 5 animals), moderate (5.19–5.73, 8 animals), and severe group (5.99–6.83, 4 animals). Sham operated animals (*N* = 10) had HBWR in the range 2.26–3.31; however, even the mild ACF group had significantly enlarged hearts due to increase in weight of both ventricles. Lung weights were also increased in the ACF group, especially in severe cases (Table 1), but there was neither ascites nor hydrothorax present in any of the animals sampled. Lung weight adjusted to body weight—a marker of heart failure development—increased continually with increasing heart weight. Both left and right ventricular weight was increased, in absolute values as well as when indexed for body weight. The ratio of right to left ventricular weight, indicating relative RV hypertrophy, was increased in the severe group.

Correlations between different parameters were analyzed within the ACF group. Not surprisingly, the HBWR correlated well with both the left (*R* = 0.66, *p* = 0.004) and right (*R* = 0.79, *p* = 0.0002) ventricular weight, making any of these parameters a good indicator of heart failure severity. All these parameters showed good correlation (*R* = 0.5–0.7, *p* < 0.05) with the lung weight in both absolute numbers and when indexed with body weight. However, body weight was not associated with the severity of cardiac hypertrophy, similar to our previous findings (Melenovsky et al., 2012). Left ventricular weight was closely correlated with right ventricular weight (*R* = 0.76, *p* = 0.0004), indicating biventricular hypertrophy. Heart rate, QRS, QT, and QT(c) durations were quite independent of phenotype severity (or showed no changes at all). Prolongation of some ECG intervals (PR, QRS) in the setting of ventricular hypertrophy is not surprising, since the impulse has greater distance to travel (Benes et al., 2011b), and is not incompatible with normal conduction velocity. APD50 and APD90 quite logically correlated well with each other (*R* = 0.75, *p* = 0.0006). APD50 was increased in the whole ACF group (Figure 1) with no significant changes between the subgroups; when these were tested individually against sham animals, only the values from the moderate group reached statistical significance (Table 1).

Interesting associations became apparent when the analysis was performed based on division of compensated/decompensated heart failure based upon lungs to body weight ratio below/over 5, resulting in 10 non-failing and 7 failing hearts within the ACF group. According to this division, a significantly increased right to left ventricular weight ratio was observed in the failing group, and there was a mild trend toward increased body weight, explainable by increased fluid retention. Even such division confirmed that some parameters (QT interval, anisotropy) showed no differences between groups.

ECG analysis showed a significant prolongation in P wave duration in all ACF subgroups (+24%), and PR interval (+11–23%) without any differences in heart rate compared to controls (Table 1). QRS duration was likewise prolonged (on average +17%, slightly more in the severe cases) in the ACF group, but was constant in the three ACF sub-groups. QT and QT(c) intervals were, on the other hand, unchanged. Q, R, and S wave amplitude was increased, and there was a significant ST segment depression. T wave amplitude was significantly decreased, as the T wave was frequently inverted (Figure 1).

Figure 2 shows typical examples of activation maps from different groups. The animals with mild left ventricular hypertrophy had a trend toward increase in both longitudinal (along the myocyte long axis, CV max) and transverse (perpendicular to this, CV min) conduction velocity, which reached statistical significance for the transverse velocity (Table 1). There was a bell-shaped relationship, with the CV min values in the most severe group declining (although insignificantly) below the sham animals. HBWR correlated inversely with minimal and maximal conduction velocities (Figure 2). In the most severe group, there were further irregularities of conduction, with areas of block present in two of four hearts analyzed (Figure 2, bottom map panels).

**Figure 3:**
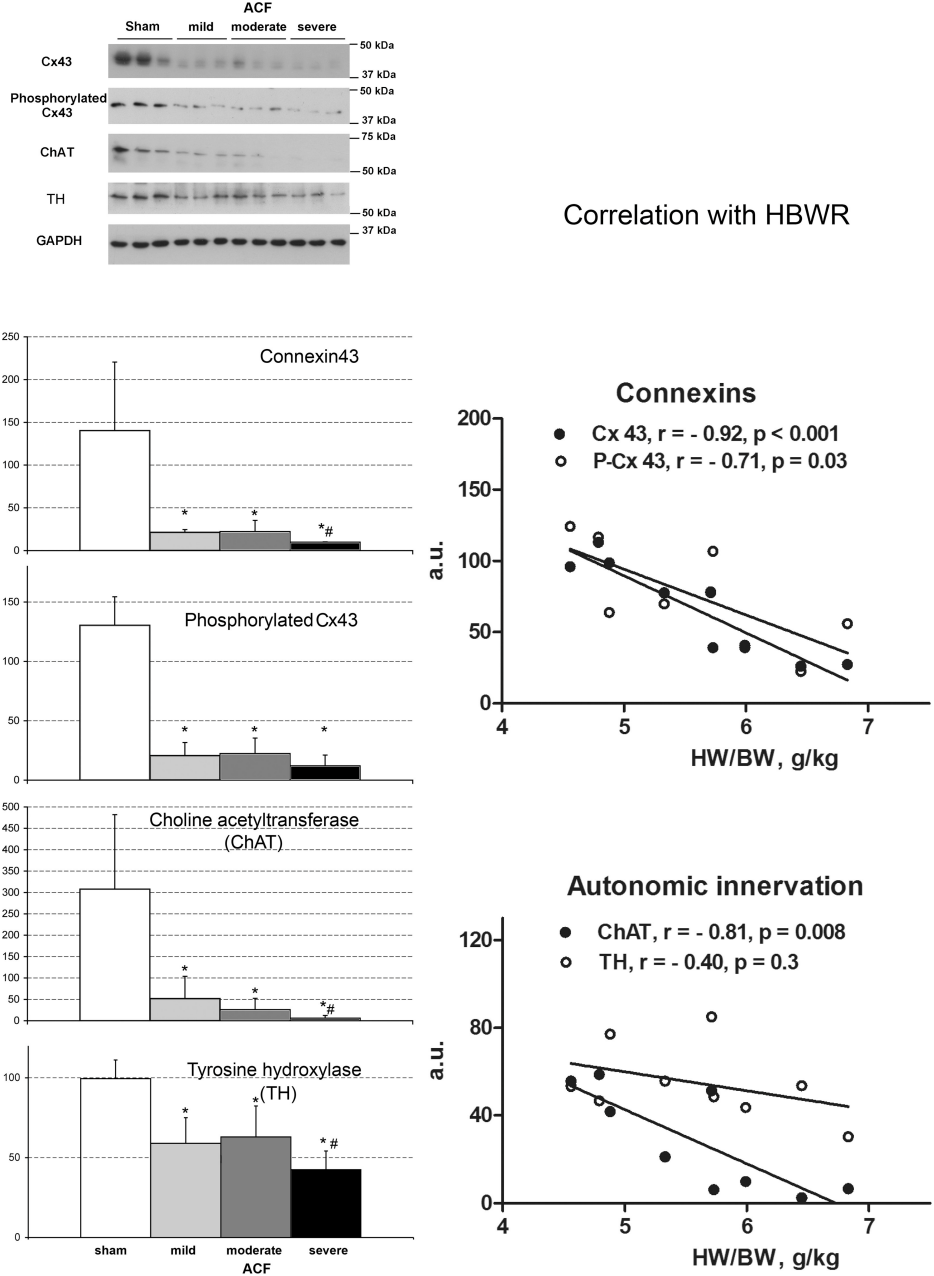
**Changes in connexin expression and autonomic innervation markers after ACF**. Values are means ± SD, *N* = 3 samples per group, ^*^p < 0.05 vs. sham, #*p* < 0.05 vs. less severe ACF groups (*t*-test). ChAT, choline acetyltransferase, TH, tyrosine hydroxylase. Graphs show correlation analysis of connexins and autonomic innervation markers with phenotype severity. Amounts of all proteins detected by Western blot decrease with increasing cardiac mass. *N* = 9 samples from the ACF group (3 for each sub-group), r, Pearson's correlation coefficient. Line represents linear regression.

**Figure 4:**
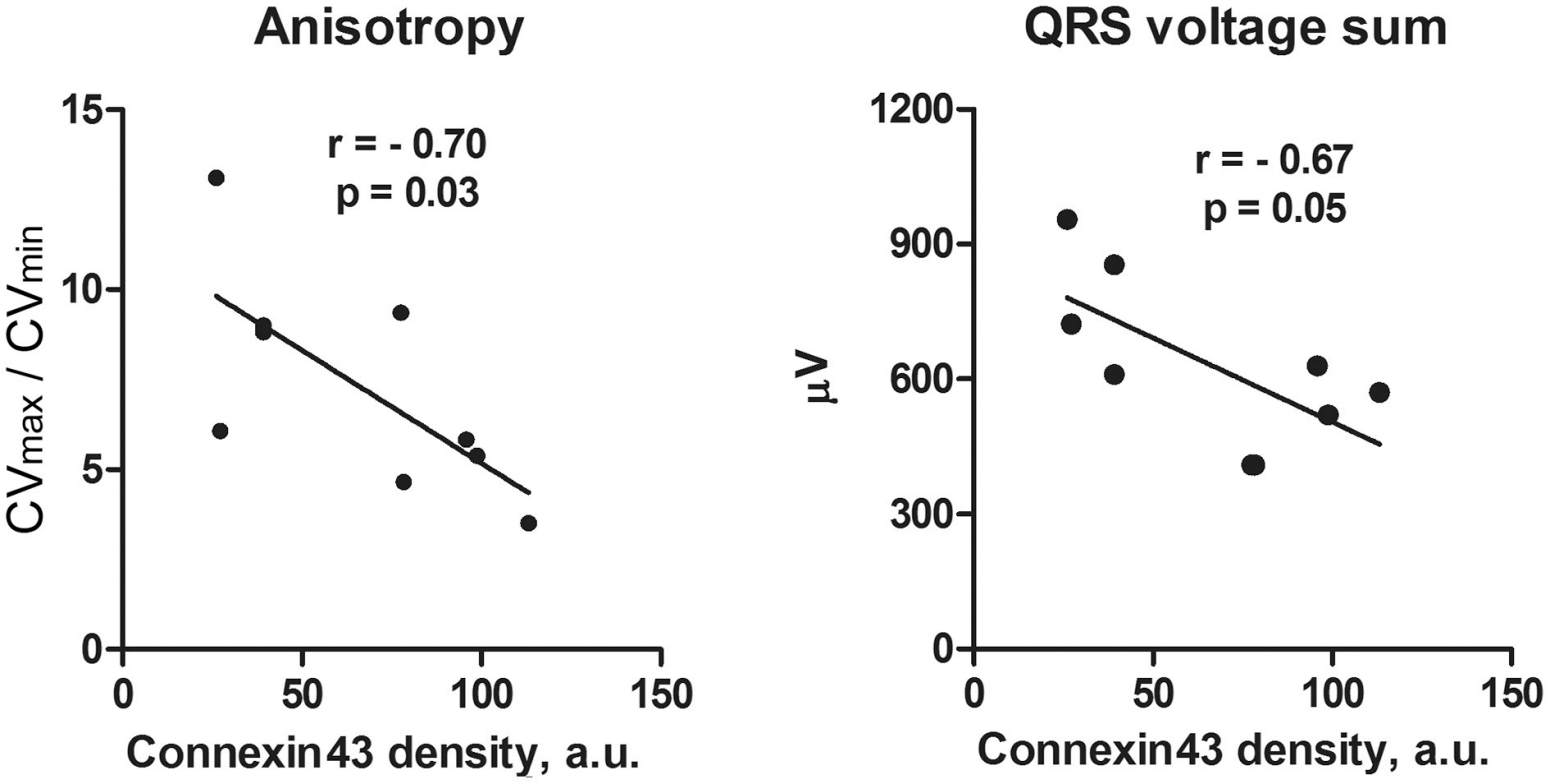
**Correlation analysis of anisotropy of conduction (A) and QRS voltage (B) with connexin43 amount in the ACF rats**. With decreasing connexin43 expression that represents increasing phenotypic severity within the ACF group, there is an increase in conduction anisotropy and amplitude of QRS voltage. *N* = 9 samples from the ACF group (3 for each sub-group), r, Pearson's correlation coefficient. Line represents linear regression.

Western blotting (Figure 3) detected a significant decrease in both total and phosphorylated connexin43 protein in the ACF hearts, consistent with the previous results (Benes et al., 2011a). Phenotype severity (HBWR) inversely correlated with both connexin43 or P-connexin43 densities with the most enlarged hearts showing the most pronounced decrease (Figure 3). Total connexin43 amount was strongly negatively correlated with biventricular weight (*R* = −0.92, *p* = 0.0004); interestingly, less with the left (*R* = −0.55, *p* = 0.1177) and more with the right (*R* = −0.77, *p* = 0.0277) ventricular weight. Total connexin43 was also negatively correlated with anisotropy ratio (*R* = −0.70, *p* = 0.0348) and QRS sum amplitude (*R* = −0.67, *p* = 0.05; Figure 4). This suggests strongly a critical role of this gap junctional protein in myocardial conduction properties, together with changes in ion channels responsible for the changes in APD. Similar behavior was observed for the phosphorylated connexin. Interestingly, correlation coefficient between these two isoforms was only 0.63 (*p* = 0.06), suggesting that the amount of connexin43 and its phosphorylation might be regulated by separate mechanisms. The ratio of these isoforms mildly (0.49, *p* = 0.1771) correlated with the QRS duration.

To assess the role of autonomic signaling, we measured the expression of choline acetyltransferase (ChAT) and tyrosine hydroxylase (TH) as a proxy for cholinergic (vagal) and adrenergic innervation, respectively. We found a clear decrease in both ChAT and TH, correlating with the phenotypic severity (Figure 3). ChAT was strongly negatively correlated with HBWR (*R* = −0.81, *p* = 0.0081) and loosely (*R* = 0.52, *p* = 0.1496) with QRS duration. It paralleled the changes in minimal conduction velocity (*R* = 0.85, *p* = 0.003), maximal velocity (*R* = 0.57, *p* = 0.1054), APD50 (*R* = 0.76, *p* = 0.0163), but with APD90 the correlation coefficient was only 0.1 (*p* = 0.788). The values went also along with the changes in connexin43 (*R* = 0.93, *p* = 0.0003) and its phosphorylated isoform. It showed a strong negative correlation (*R* = −0.83, *p* = 0.005) with lungs to body weight ratio, suggesting its expression was decreased especially in the severely failing hearts.

**Figure 5:**
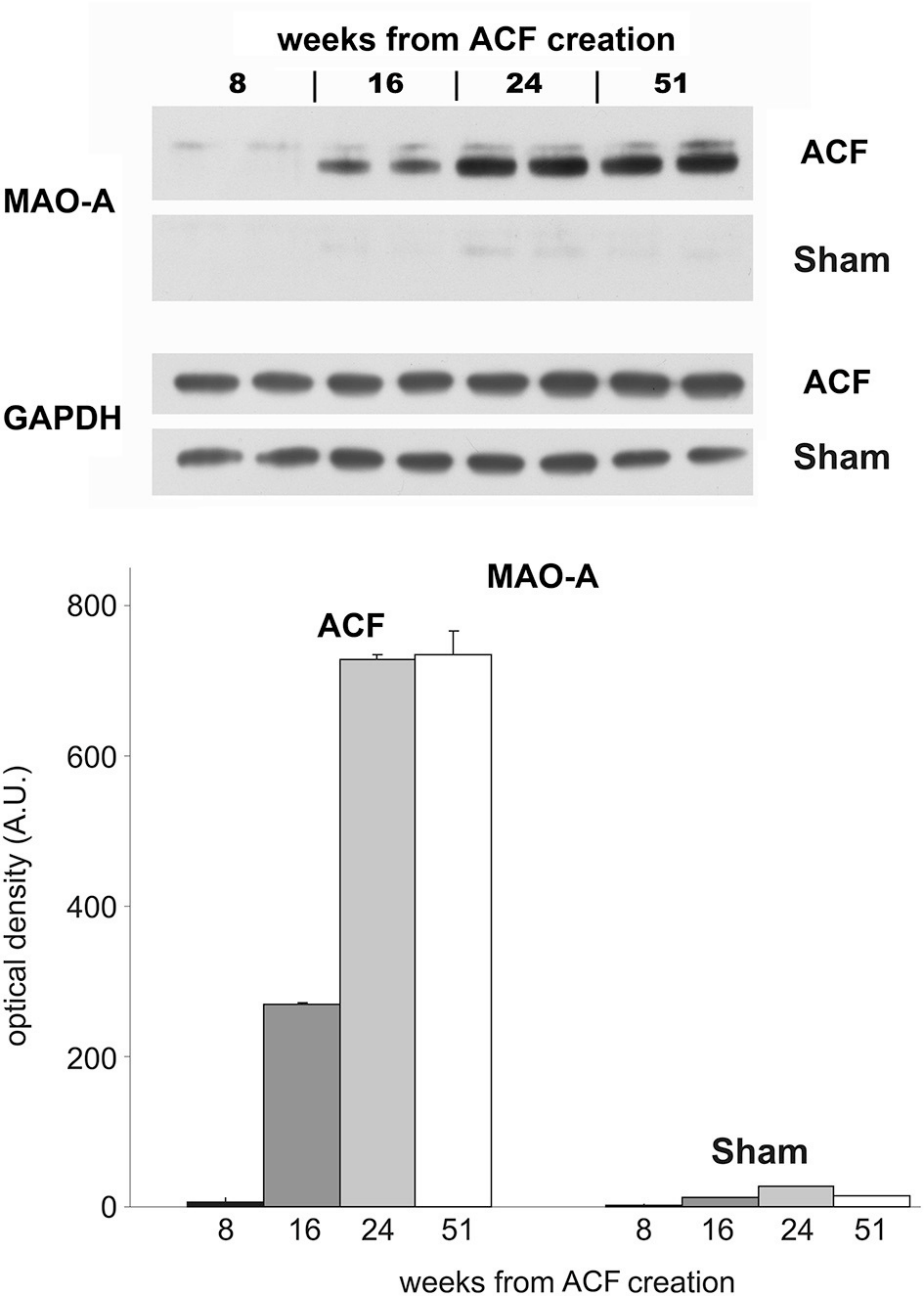
**Longitudinal changes in MAO expression in the ACF model**. Western blot was performed with samples from left ventricles of animals with ACF and sham-operated animals (animals sacrificed 8, 16, 24, and 51 weeks after creation of ACF). Each sample represents a pooled tissue homogenate from five animals from the individual group. Samples were loaded in duplicates on the 10% minigels. As a primary antibody rabbit anti-monoamine oxidase A was used (MAO-A, 1:333, Sigma-Aldrich, MO, USA).

Since the role of adrenergic stimulation is well established in heart failure, we investigated separately the time course of adrenergic signaling in a parallel longitudinal study, using the catecholamine degradation enzyme monoamine oxidase (MAO) expression as a proxy (Figure 5). During the course of development of eccentric hypertrophy after creation of ACF, there was a steady increase in the adrenergic activity, evidenced by increased levels of MAO detected by Western blotting. These changes reached a plateau at 24 weeks, suggesting that near maximal adaptation was already in place during our sampling. Increased adrenergic stimulation can be viewed as an adaptive measure to increase myocardial performance during increased functional demands, despite blunting of the beta-adrenergic signaling reported in heart failure (Aiba and Tomaselli, 2010).

## Discussion

### Electrophysiological changes

Despite a massive increase in heart weight accompanied by a drastic connexin43 downregulation, the conduction velocity was decreased only mildly and only in the most severe group. In contrast, the mildly hypertrophied hearts showed instead an increase in impulse conduction velocity. This goes against the common notion that cardiac hypertrophy is associated with slowing of the conduction velocity, which is considered to be a major factor predisposing the heart to arrhythmias. We do not view our results as contradictory, since simple elongation of the myocytes is likely, according to the cable theory, to increase conduction velocity, and the mild and moderate groups present a compensated stage of hypertrophy without overt failure. In the severe cases we observed return to normal values and further decrease in gap junction protein connexin 43 (Table 1, Figure 3). Indeed, the most severely affected animals were dying of sudden death in a longitudinal survival study in this model (Melenovsky et al., 2012), suggesting that only the severe hypertrophy combined with heart failure constitute the arrhythmogenic substrate, consistent with a recent review of clinical studies (Stevens et al., 2013). Observations similar to ours were made in a rabbit pressure-overload hypertrophy model by Wiegerinck et al. (2006), who demonstrated increased longitudinal conduction velocity despite lower levels of connexin43. Mathematical modeling in this interesting study showed that increased conduction velocity could be explained by cell enlargement (especially elongation) and by low levels of fibrosis. Increased conduction velocity may not fully compensate the ventricular hypertrophy, resulting in a significant QRS prolongation reported in some failing hearts (Anderson et al., 1993; Winterton et al., 1994; Cooklin et al., 1998). It is difficult to quantify from our data the relative contribution of increased (doubled) distance the impulse must travel (Benes et al., 2011b) and changes in CV–QRS and PR interval duration. The conduction system shows a rich autonomic innervation, and we found a significant reduction in markers of both adrenergic and cholinergic innervation; however, since our ECG measurements come from the anesthetized animals, these data should not be over-interpreted. Considering further the ambiguities in determining ECG intervals in hypertrophied hearts, we are planning future *in vitro* studies using plunge electrodes and optical mapping of wedge preparation to further dissect the transmural conduction through the Purkinje system and working myocardium. The model of volume overload hypertrophy (Melenovsky, 2013) is unique by lowering of collagen content in the myocardium accompanying dilation (Ryan et al., 2007). Development of end-stage heart failure in this model is then associated with increased collagen production, explaining the drop in conduction velocity below the control levels in the most severely hypertrophied hearts (Hutchinson et al., 2010, 2011).

Previously conducted experimental studies of impulse propagation in cardiac muscle used canine or sheep myocardium (Kadish et al., 1986; Delgado et al., 1990). Most of the studies conducted on rat myocardium was done on tissue cultures of myocyte monolayers (Fast et al., 1996; McSpadden et al., 2009), which might behave quite differently than the native tissue with fibrous scaffolding, blood vessels, and other sources of heterogeneity. In these studies the anisotropy ratio was approximately around 2–3, which is much less than in our study. Some of the previous studies on mathematical models of the native myocardium showed much higher anisotropy values, with longitudinal-to-transverse velocity ratios of 5.7 (Leon and Roberge, 1991) and 9.6 (Muller-Borer et al., 1994), which much more correlate with our findings. The reason for such high anisotropy can be also partly artificial—the velocities are calculated from 2D image of epicardial signal spreading from the center of the left ventricle. Given the curvature of the heart, crowding of the isochrones can occur in the transverse direction, which can lead to an artificial slowing of the CV min.

This study attempts to dissect the changes in established markers of cardiac hypertrophy and failure during the progression from compensated to decompensated stage. ECG changes in the ACF animals confirmed increased cardiac mass (P and QRS wave amplitude) and suggest prolonged atrial and ventricular conduction time, which could be well due to the larger size of these compartments. ACF-induced hypertrophy and heart failure were associated with a mild APD prolongation (Figure 1), which was significant only in the early phase (APD50). Unfortunately, residual motion artifacts during the repolarization phase prevented reliable estimation of action potential dispersion, which would be also an interesting parameter expected to be altered in hypertrophied hearts. This might suggest alterations in ion channels (Shah et al., 2005; Aiba and Tomaselli, 2010). Some of these changes might be also due to alterations in calcium cycling, which is known to occur in the settings of heart failure (Ai et al., 2005; Guggilam et al., 2012). Conduction anomalies (mostly slowing) observed in non-ischemic dilated cardiomyopathy were reviewed by Akar and Tomaselli (2005) and include changes in myocyte excitability (sodium and potassium channels), extracellular matrix (fibrosis, mostly absent in this model), and cell coupling (connexins). Tissue architecture (cell shape and size) is also an important factor, and there is an agreement that anisotropy of conduction is preserved in this setting, corresponding well with our present data. Expression of numerous ion channels such as sodium channel NaV1.5 (de la Rosa et al., 2013), potassium channels or NaH exchanger is often implicated in arrhytmogenesis in cardiac hypertrophy and/or failure (Stevens et al., 2013) and detailed analysis at the molecular and functional level together with temporal dynamics would require numerous further experiments using alternative techniques (RT-PCR, patch clamp, isolated myocytes).

### Changes in gap junctional proteins

Our findings of connexin43 downregulation and hypophosphorylation correspond well with our previous study (Benes et al., 2011a), that of Guggilam and colleagues in the ACF model (Guggilam et al., 2012) as well as some of the others including different animal models (Ai and Pogwizd, 2005; Akar et al., 2007). However, the role of connexin43 cellular distribution is less well understood, as we did not previously observe any significant lateralization on the cell membrane, in contrast with others (Akar et al., 2004). Considering the complexity of tissue architecture and likely influence of sampling location and exact analysis method, the *in vivo* studies could be expected to differ in details. This complexity turned our attention instead to possible relationship between more precisely quantifiable parameters like the total amount and protein phosphorylation detected by Western blot and degree of ventricular hypertrophy; for the first time, we report here a clear and often significant trend of more severe changes in advanced hypertrophy and heart failure.

### Autonomic innervations

We observed a large drop in the amount of choline acetyltransferase, a marker of cholinergic signaling, which was most pronounced in the severe ACF group. Most cholinergic terminations are supposed to be present around the coronary arteries. Our findings agree with other reports indicating that parasympathetic ganglia are present in the ventricles, connecting to relatively dense parasympathetic innervation of ventricular muscle that may modulate excitability and propensity to arrhythmias at the local level, independently of activation of sympathetic fibers (Coote, 2013). Acetylcholine can be synthesized in cardiomyocytes as well—this non-neuronal source may boost parasympathetic cholinergic signaling to counterbalance sympathetic activity (Roy et al., 2013). Indeed, pharmacological M2 muscarinic receptor activation was proposed to present an alternative to beta blockers (Rauch and Niroomand, 1991). On the other hand, M2 antagonist cisatracurium and atropine were demonstrated to suppress vagally-mediated action potential shortening and prevent atrial arrhythmias (Patterson et al., 2008).

Despite of global sympathetic activation, severe heart failure is simultaneously characterized by loss of cardiac sympathetic neurons and noradrenergic terminals, leading to myocardial norepinephrine depletion that likely further contributes to pump failure (Himura et al., 1993). In line with this previous observation, our study documented inverse relation between abundance of sympathetic marker TH and left ventricular hypertrophy severity or degree of congestion. We have verified the results from the western blot by immunohistochemistry performed on sister sections of sham and ACF (21 weeks) animals used in a previous study (Benes et al., 2011a). Western blotting represents a more objective measure, since the immunostaining is highly non-uniform within the ventricular wall. We observed that sympathetic and parasympathetic denervation goes in parallel, and our study does not support presence of cholinergic transdifferentiation as suggested in another rodent HF model (Kanazawa et al., 2010). Decreased sympathetic nerve density and signaling is in agreement with blunting of the beta-adrenergic signaling reported previously in this type of heart failure model (Aiba and Tomaselli, 2010).

It would be interesting to explore further the physiological consequences of altered autonomous innervation; however, truly physiologically relevant studies would need to be performed in active animals using telemetry, which is beyond the scope of the present study. Analysis of parameters such as heart rate variability, decrease of which is a sign of cardiac distress, would then be possible; it could provide further insight beyond the resting heart rate of anesthetized animals, which was not significantly changed in our study (Table 1).

### Prognostic markers of heart failure?

Longitudinal study of rats with ACF showed that 82% of operated animals develop heart failure, and 72% die within 1 year (Melenovsky et al., 2012). Prior to death due to heart failure, there was first a mild increase in body weight due to water retention, followed by a drop likely due to anorexia. Most of the symptoms (e.g., increased lung weight) are of the left ventricular failure; we believe it is due to the fact that the left ventricle is primarily constructed as a pressure pump, tolerating well pressure overload (such as in banding models or systemic hypertension), unlike the right ventricle, which is design as a volume pump tolerating well increased preload, but not afterload (Hutchins et al., 1978; Pesevski and Sedmera, 2013). Since most sudden deaths (likely due to arrhythmias) occurred in the most enlarged hearts, we sought for other parameters that could predict or explain these deaths. We can divide the measured parameters into several groups according to their behavior during the development of eccentric ventricular hypertrophy and heart failure. In the first group, there is a quantitative change in hypertrophy independent of its magnitude. These are the markers of cardiac hypertrophy and include most of the ECG parameters—P wave amplitude and duration, PR interval duration, QRS interval duration, and ST segment depression. They reflect adaptive changes such as myocardial hypertrophy as well as ischemia. Another electrophysiological parameter without a clear correlation with phenotype severity is prolongation of action potential duration. The second group is similar in showing considerable differences between normal hearts and hypertrophied/failing ones, but with a significantly higher change in more hypertrophied group. These are mostly gross weight parameters, which are direct indicators of the level of ventricular hypertrophy, but also signs of heart failure such as lung weight (in extreme cases leading to hydrothorax, and ascites). On the electrophysiological level, this was represented by abnormal conduction including blocks in the severe group (Figure 2). These changes were reflected on the protein level by gradual decrease of connexin43 protein, a trend in the phosphorylated isoform, and dose-response in TH and choline acetyltransferase expression. All these parameters are potentially useful as indicators of transition from compensated to decompensated stage of hypertrophy and imminent death, either from heart failure, or arrhythmogenic.

## Conclusions and perspectives

In this study, we explored changes in ventricular myocardium in the settings of volume-overload heart failure as a potential substrate for arrhythmias. ECG changes were consistent with eccentric hypertrophy. Electrophysiologically, we observed an increase in minimal conduction velocity in the mild ACF group and partial conduction blocks in the severe group, while there were no significant changes in maximal conduction velocity among groups. Both maximal and minimal conduction velocity inversely correlated with the degree of cardiac hypertrophy, and APD50 was mildly prolonged. We confirmed a significant decrease in connexin43 protein expression and phosphorylation, with further accentuation of these changes in the most severely hypertrophied hearts. This was accompanied at the biochemical level by decreased cholinergic and increased adrenergic neurotransmitter expression, showing the role of the autonomic innervation in adaptation to increased volume loading. Additional studies might deal with the temporal dynamics of expression and function of the ion channels, and telemetry recordings could document and quantify the actual *in vivo* frequency of arrhythmias. Future attention will be focused on the right ventricle, which shows even higher degree of hypertrophy in this model. Furthermore, remodeling of the atria could be also analyzed in the context of arrhythmogenesis in heart failure. These findings could direct our future therapeutic strategies in similar clinical settings.

## Author contributions

Designed the study: JB, DS, VM, JiP. Performed experiments: DS, JN. Analyzed data: JB, JaP, KS. Interpreted data: JiP, DS, VM. Wrote and read the paper: DS, JN, JB, JaP, JiP, KS, VM.

## Funding

This work was supported by BIOCEV-Biotechnology and Biomedicine Center of the Academy of Sciences and Charles University in Vestec (CZ.1.05/1.1.00/02.0109), from the European Regional Development Fund. MZ CR project for the development of research organization 00023001 (IKEM)—institutional support and by EU-funded Operational Program Prague-Competitiveness; project “CEVKOON” (#CZ.2.16/3.1.00/22126); by Ministry of Education (PRVOUK P35/LF1/5 to DS, P24/LF1/3 and SVV 260265/2016 to JP, SVV-2013-266509 and KONTAKT-II LH12052 to VM); Academy of Sciences of the Czech Republic (RVO: 67985823 to DS); Grant Agency of the Czech Republic (P302/11/1308 and 13-12412S to DS and 15-14200S to JP and VM) and Agency for Healthcare Research (AZV 15-27682A to VM and 15-27735A to JN); and Charles University in Prague, First Faculty of Medicine training fellowship program (to JB).

### Conflict of interest statement

The authors declare that the research was conducted in the absence of any commercial or financial relationships that could be construed as a potential conflict of interest.
